# Diagnostic value of combined FVC%/DLCO% and echocardiography in connective tissue disorder‑associated pulmonary hypertension

**DOI:** 10.3892/mi.2024.132

**Published:** 2024-01-05

**Authors:** Huimin Shi, Pengfei Gao, Huijin Liu, Jie Su, Xuegai He

**Affiliations:** The First Affiliated Hospital of Henan University of Science and Technology, Luoyang, Henan 471003, P.R. China

**Keywords:** connective tissue disorder-associated pulmonary hypertension, forced vital capacity/diffusing capacity of the lungs for carbon monoxide, echocardiography, plasma brain natriuretic peptide, right heart catheterization

## Abstract

The main objective of the present study was to investigate whether forced vital capacity (FVC)%/diffusing capacity of the lungs for carbon monoxide (DLCO)% can be used to predict the presence of pulmonary hypertension (PH) in connective tissue disorders (CTDs). For this purpose, a total of 53 individuals who were diagnosed with CTDs and had undergone right heart catheterization between July, 2019 and July, 2022 were included in the present study. Based on the mean pulmonary artery pressure (mPAP) measured during right heart catheterization, the participants were divided into the PH and non-PH groups. The differences in demographic characteristics, including sex, age, body mass index, smoking index, FVC%/DLCO% and pulmonary artery systolic pressure (PASP) were determined by echocardiography; moreover, the 6-min walk distance, plasma brain natriuretic peptide (BNP) levels, white blood cell count, red blood cell distribution width, erythrocyte sedimentation rate and C-reactive protein levels were compared between the two groups to identify independent predictors of PH. The independent predictors were subsequently evaluated for their correlation with mPAP to assess their predictive value for PH. FVC%/DLCO%, echocardiographic PASP, and plasma BNP levels were identified as independent predictors of PH. FVC%/DLCO% and echocardiographic PASP exhibited a significant correlation with mPAP, while the correlation between plasma BNP and mPAP levels was not statistically significant. The area under the curve (AUC) value for FVC%/DLCO% alone in predicting PH was 0.791, with an optimal diagnostic threshold of 1.35, a sensitivity of 0.794 and a specificity of 0.789. The AUC for echocardiographic PASP alone in predicting PH was 0.783, with an optimal diagnostic threshold of 39.5 mmHg, a sensitivity of 0.794 and a specificity of 0.684. When combined, the AUC of the two factors in predicting PH was 0.872, with a sensitivity of 0.941 and a specificity of 0.684. Collectively, the data of the present study indicate that FVC%/DLCO% may be used as a predictive factor for CTD-PH, and its combined application with echocardiographic PASP measurement may provide additional evidence for the clinical diagnosis of CTD-PH.

## Introduction

Pulmonary hypertension (PH) is a cardiopulmonary disease characterized by increased pulmonary vascular resistance, leading to elevated pulmonary arterial pressure, progressive right heart failure and ultimately, death, caused by various etiologies (1,2). The ‘2022 ESC/ERS Guidelines for the diagnosis and treatment of pulmonary hypertension’ define a hemodynamic criterion for diagnosing PH as a mean pulmonary artery pressure (mPAP) >20 mmHg, measured during resting right heart catheterization (3).

Connective tissue disorder (CTD)-associated PH (CTD-PH) is the second most common cause in group 1 arterial PH, following idiopathic pulmonary arterial hypertension (4). PH is a severe and life-threatening complication of CTDs, primarily occurring in systemic sclerosis, systemic lupus erythematosus, mixed connective tissue disease and less frequently, in rheumatoid arthritis, inflammatory myopathies and Sjögren's syndrome (5,6). Among patients with CTDs, those with concurrent PH have a significantly worse prognosis and lower survival rates compared to those without PH (7). Therefore, the early detection of PH in CTDs and proactive intervention are of utmost importance.

As mentioned previously, in the ‘2022 ESC/ERS Guidelines for the diagnosis and treatment of pulmonary hypertension’, right heart catheterization is considered to be the gold standard for the diagnosis of PH (3); however, it is an invasive procedure associated with potential complications, risks and technical difficulties, limiting its clinical application. Transthoracic echocardiography with Doppler is recommended as the primary non-invasive screening and evaluation method for PH (3). However, there is a certain degree of mismatch between the pulmonary artery systolic pressure (PASP) obtained through echocardiography and the mPAP measured by right heart catheterization, leading to potential underdiagnosis or misdiagnosis, notably when Doppler envelope quality is fair or poor (8). Therefore, there is a requirement to establish a simpler non-invasive tool for identifying PH. Previous studies have reported a correlation between the forced vital capacity (FVC)%/diffusing capacity of the lungs for carbon monoxide (DLCO)% and the presence of PH in patients with systemic sclerosis (9-11). Recently, another study indicated that FVC%/DLCO% could identify PH associated with chronic obstructive pulmonary disease and predict the 5-year all-cause mortality of patients with chronic obstructive pulmonary disease (12). Therefore, the primary focus of the present study was to investigate whether FVC%/DLCO% can be used to predict PH in a range of CTDs.

## Materials and methods

### Study subjects

The present study included consecutive patients who underwent right heart catheterization at the Respiratory and Critical Care Medicine Center, The First Affiliated Hospital of Henan University of Science and Technology between July, 2019 and July, 2022 and were diagnosed with CTD and suspected concurrent PH by the rheumatology and immunology specialists. The inclusion criteria required adherence to the following conditions: i) A diagnosis of relevant CTDs, including systemic lupus erythematosus (13), rheumatoid arthritis (14), inflammatory myopathies (15), systemic sclerosis (16) and Sjögren's syndrome (17), based on the diagnostic criteria of the American College of Rheumatology/European League Against Rheumatism (13-17) and based on the criteria by Sharp *et al* (18) for mixed connective tissue disease; ii) transthoracic Doppler ultrasound indicating PASP >30 mmHg. The exclusion criteria were as follows: i) An age <18 or >70 years; ii) contraindications for pulmonary function testing, such as pneumothorax, aortic aneurysm, recent myocardial infarction, active pulmonary tuberculosis, or inability to cooperate with the test; iii) contraindications for right heart catheterization, such as bleeding tendency, severe arrhythmia or acute infection; iv) incomplete clinical data. Finally, a total of 53 patients were included in the present study and were categorized into two groups based on a mPAP threshold of 20 mmHg. The groups were the following: PH group (34 cases) and non-PH group (19 cases). The present study was approved by the Ethics Committee of the First Affiliated Hospital of Henan University of Science and Technology (2019-03-K0027) and all participants provided written informed consent.

### General data collection

The detailed records of demographic data, clinical information and laboratory test results were collected for all participants. This included sex, age, body mass index, smoking status, transthoracic echocardiography PASP, FVC%/DLCO%, 6-min walk distance, plasma brain natriuretic peptide (BNP) levels, white blood cell count, red cell distribution width, erythrocyte sedimentation rate and C-reactive protein levels.

### Pulmonary function test

Spirometry was performed using the Jaeger lung function analyzer (SpringDe Electronic Technology Co., Ltd.) to assess the patient's pulmonary ventilation and diffusion function. Forced expiratory volume in one second (FEV1), FVC, FEV1% FVC, FVC%, DLCO% and other measurements were performed in all subjects participating in the study in accordance with the recommendations of the American Thoracic Society and the European Respiratory Society (19,20). The ‘single breath method’ was used for DLCO measurements and DLCO measurements were corrected for serum hemoglobin, as previously described (21).

### Echocardiograms

Resting two-dimensional transthoracic echocardiography was performed using standard techniques. The transtricuspid pressure gradient was calculated using the modified Bernoulli equation (4v2), where ‘v’ is the maximum velocity of the tricuspid valve regurgitant jet. The right atrial pressure (RAP) was estimated by the respiratory variation in the diameter of the inferior vena cava. The right ventricular systolic pressure (RVSP) was calculated by addition of the transtricuspid pressure gradient to the RAP estimate (22). PASP was approximately equal to RVSP.

### Right heart catheterization

All enrolled patients underwent right heart catheterization using a Swan-Ganz catheter (Edwards Lifesciences Corp.). The catheter was inserted via the right femoral vein using the Seldinger technique. Once a successful puncture was achieved, a guidewire and sheath were inserted. The distal extension tube of the catheter was connected to the pressure transducer; the thermistor connector was connected to the sensor and the inflation valve of the balloon to the inflation syringe (1.5 ml). The position of the catheter tip was determined based on the changes in the pressure waveform. The catheter was advanced until a wedged waveform was observed, indicating pulmonary artery wedge pressure. Pressure measurements were recorded at various locations including the pulmonary artery, right ventricle, right atrium and vena cava during quiet expiration.

### Statistical analysis

Statistical analysis was performed using SPSS 27.0 software. Continuous variables are expressed as the mean ± standard deviation. The t-test was used to compare continuous variables between two groups. Categorical variables are presented as numbers and percentages and the χ^2^ test was used to compare the different categorical variables between groups. Logistic regression analysis was performed to identify independent predictive factors for significant differences. Pearson's correlation analysis was conducted to assess the correlation between independent predictive factors and mPAP. Receiver operating characteristic (ROC) curve analysis was used to evaluate the diagnostic value of each predictive factor for the disease and a logistic regression model was established for joint diagnosis to explore the feasibility of disease assessment. P<0.05 was considered to indicate a statistically significant difference.

## Results

### Disease composition of CTD-PH

A total of 53 eligible patients with six types of CTDs (systemic lupus erythematosus, rheumatoid arthritis, mixed CTD, inflammatory myopathies, systemic sclerosis and Sjogren's syndrome) were included in the present study. Following right heart catheterization, they were divided into the following two groups based on a mPAP threshold of 20 mmHg: The PH group (34 cases) and the non-PH group (19 cases). Among the 34 patients with PH, 14 cases were diagnosed with systemic lupus erythematosus (41.2%), 6 cases with rheumatoid arthritis (17.6%), 6 cases with mixed CTD (17.6%), 3 cases with inflammatory myopathies (8.8%), 3 cases with systemic sclerosis (8.8%) and 2 cases with Sjogren's syndrome (5.9%; Table I).

### Comparison of population characteristics and laboratory test results

Following comparison of the population characteristics and the laboratory test results between the PH and the non-PH groups, no statistically significant differences were found as regards the parameters age, sex, body mass index, smoking index, white blood cell count, red cell distribution width, erythrocyte sedimentation rate and C-reactive protein. However, the PH group exhibited significantly higher values of FVC%/DLCO%, mPAP (which was used for grouping), echocardiographic PASP and plasma BNP compared with those noted in the non-PH group. The 6-min walking distance was significantly lower in the PH group (Table II).

### Multivariable logistic regression analysis of factors associated with pulmonary hypertension

A multivariable logistic regression analysis was conducted on the significantly different indicators (FVC%/DLCO%, PASP, plasma BNP and 6-min walking distance) to determine their independent predictive factors for PH. The results indicated that the 6-min walking distance was not an independent predictive factor for CTD-PH (P=0.470), while FVC%/DLCO%, PASP, and plasma BNP could serve as independent predictive factors for CTD-PH (Table III).

### Correlation analysis of FVC%/DLCO%, PASP, plasma BNP and mPAP

**Figure 1:**
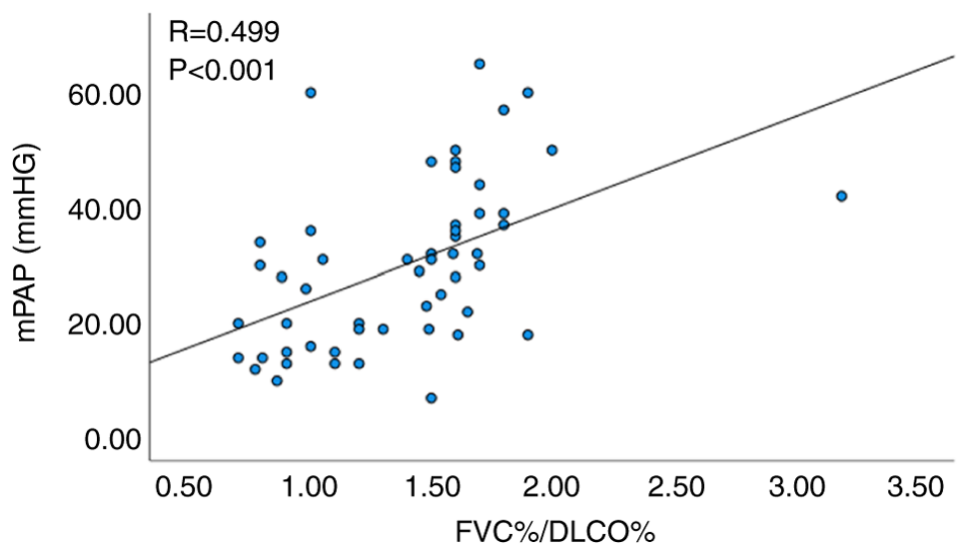
Correlation analysis between FVC%/DLCO% and mPAP. FVC, forced vital capacity; DLCO, diffusing capacity of the lungs for carbon monoxide; mPAP, mean pulmonary artery pressure.

**Figure 2:**
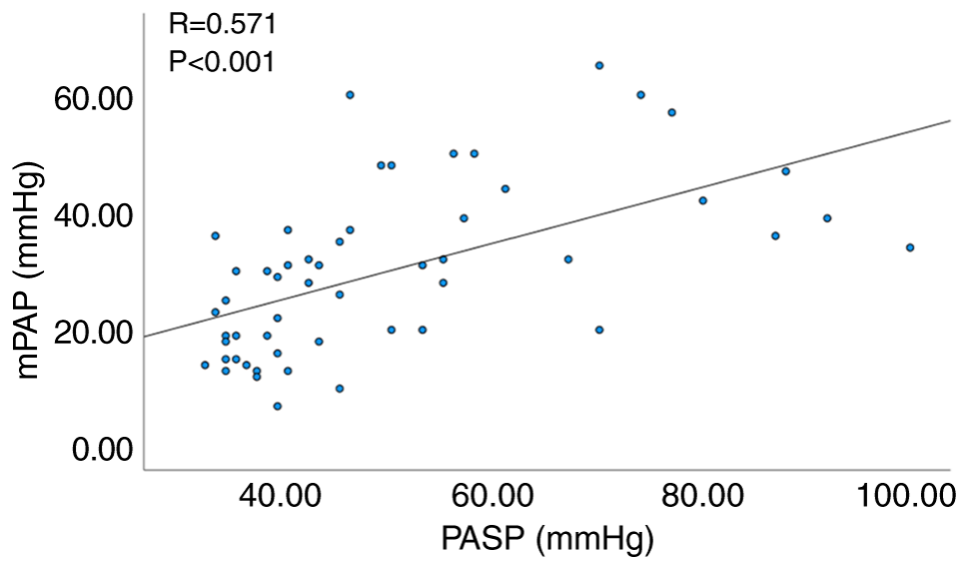
Correlation analysis between echocardiographic PASP and mPAP. PASP, pulmonary artery systolic pressure; mPAP, mean pulmonary artery pressure.

Pearson's correlation analysis was performed to examine the correlation between the selected independent predictive factors, including FVC%/DLCO%, PASP, plasma BNP and mPAP. The results indicated no statistically significant correlation between plasma BNP and mPAP (P=0.054). However, a significant correlation was noted between FVC%/DLCO% and mPAP (R=0.499, P<0.001; Fig. 1), as well as between echocardiographic PASP and mPAP (R=0.571, P<0.001; Fig. 2).

### ROC curve of FVC%/DLCO% and echocardiographic PASP for the diagnosis of CTD-PH

**Figure 3:**
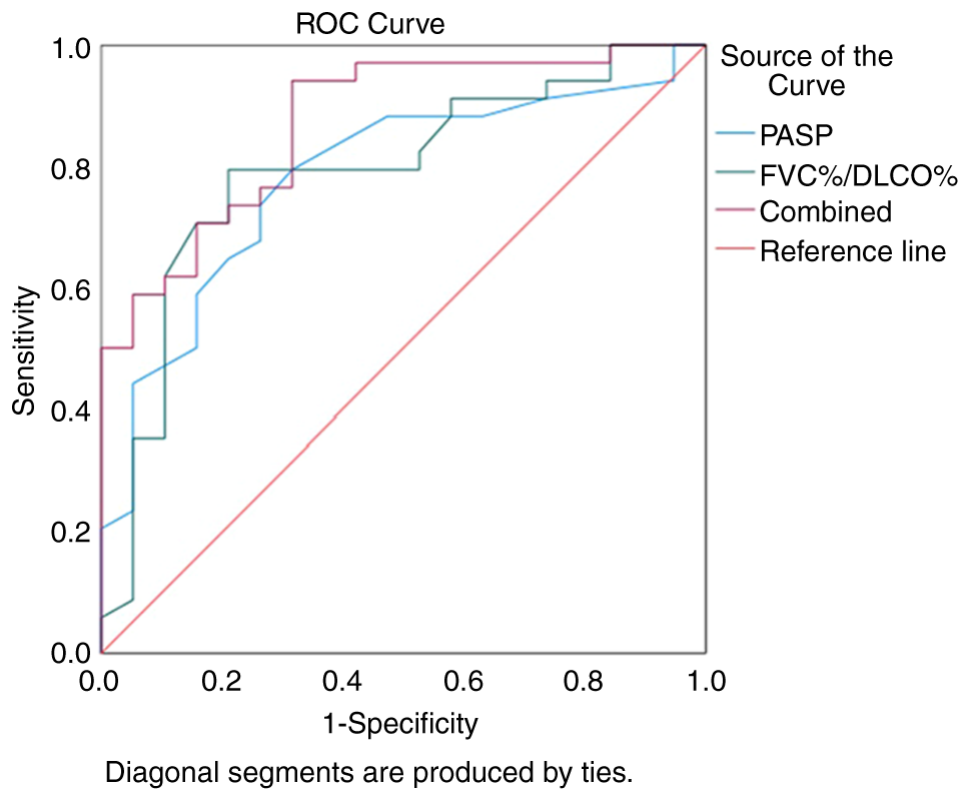
ROC curve for diagnosing PH using FVC%/DLCO% in correlation with PASP. ROC, receiver operating characteristic; PH, pulmonary hypertension; FVC, forced vital capacity; DLCO, diffusing capacity of the lungs for carbon monoxide; PASP, pulmonary artery systolic pressure.

The values of FVC%/DLCO% and PASP in diagnosing CTD-PH were determined using ROC curve analysis. The AUC for using FVC%/DLCO% alone to diagnose CTD-PH was 0.791, with an optimal diagnostic threshold of 1.35. The sensitivity was 0.794 and the specificity 0.789. The AUC for using PASP alone to diagnose CTD-PH was 0.783, with an optimal diagnostic threshold of 39.5 mmHg. The sensitivity was 0.794 and the specificity 0.684. When combining both FVC%/DLCO% and PASP for the diagnosis of CTD-PH, the AUC was 0.872, with a sensitivity of 0.941 and a specificity of 0.684. These results indicated that the combined use of FVC%/DLCO% and PASP markedly improved the diagnostic rate of CTD-PH (Fig. 3).

### Subgroup analysis excluding patients with concomitant interstitial lung disease (ILD)

**Figure 4:**
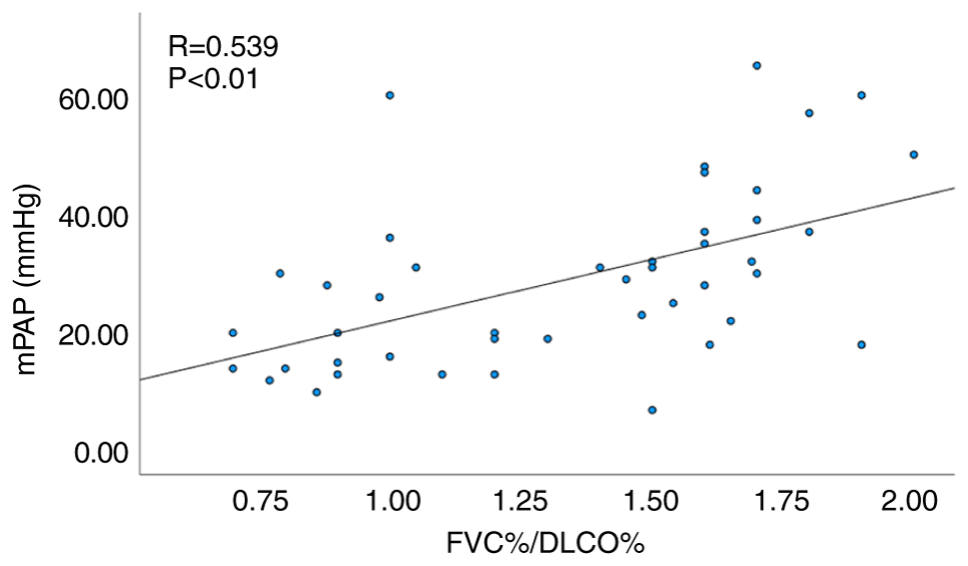
Correlation analysis between FVC%/DLCO% and mPAP in patients with CTD without interstitial lung disease. FVC, forced vital capacity; DLCO, diffusing capacity of the lungs for carbon monoxide; mPAP, mean pulmonary artery pressure.

**Figure 5:**
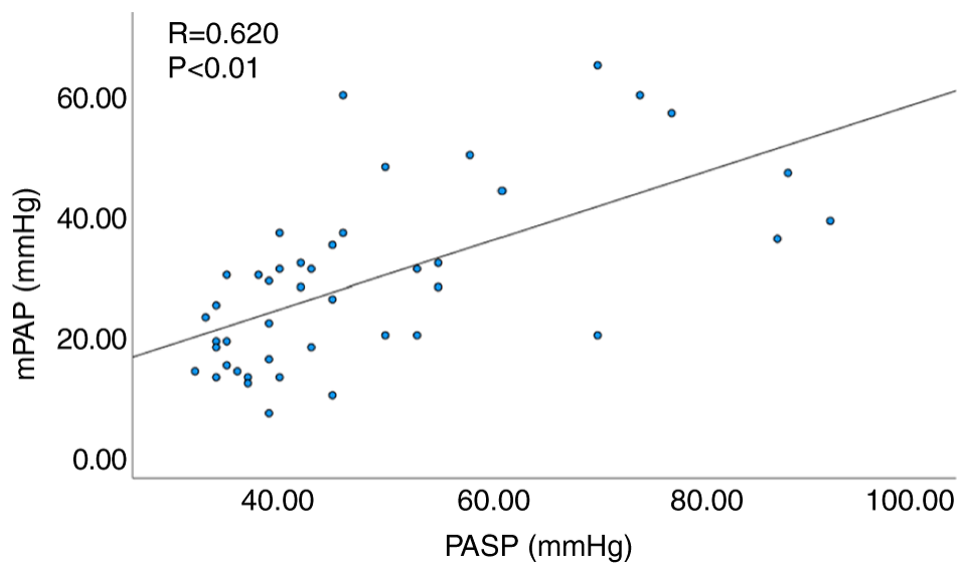
Correlation analysis between echocardiographic PASP and mPAP in patients with CTD without interstitial lung disease. PASP, pulmonary artery systolic pressure; mPAP, mean pulmonary artery pressure.

Since 9 patients presented with concomitant interstitial lung disease in the present study, the assessment of the effect of ILD was examined using a subgroup analysis. Similarly, among the 44 patients without ILD a significant correlation was noted between FVC%/DLCO% and mPAP (R=0.539, P<0.01; Fig. 4), as well as between echocardiographic PASP and mPAP (R=0.620, P<0.01; Fig. 5).

## Discussion

PH is associated with a significant impact on the quality of life of patients and high mortality rates, similar to other cardiovascular diseases (23). In a registry study on PH in the USA, CTD-PH was found to be particularly prominent in group 1 pulmonary arterial hypertension classification (24). Therefore, the timely identification and treatment of PH in patients with CTD is of utmost importance. The 2022 ESC/ERS guidelines and the Australian Scleroderma Interest Group recommend the annual screening of patients with PH with systemic sclerosis, including asymptomatic individuals, using transthoracic echocardiography, pulmonary function tests and serum biomarkers (3,25,26). However, these guidelines (3,25,26) do not specify screening for PH in other types of CTD, which may be due to the higher prevalence of systemic sclerosis-associated PH in Western countries, such as 74% in the UK and 68% in France (27,28). In Asian countries, the highest proportion of PH incidence is noted for systemic lupus erythematosus, with 58.4% in China and 35.3% in Korea (29,30). In the present study, the highest incidence of CTD-associated PH was found in systemic lupus erythematosus (41.2%), followed by rheumatoid arthritis (17.6%) and mixed connective tissue disease (17.6%). The relatively low incidence of systemic sclerosis (8.8%) in the present study aligns with data derived from large-scale studies in Asia. Therefore, active screening for PH in Asian countries, including systemic sclerosis and other types of CTDs, is necessary.

The definitive diagnosis of PH is typically based on right heart catheterization; however, this procedure is limited by its technical requirements and the inherent risks of invasive operations. The conduct of this examination for all suspected patients with PH is not practical (31). Therefore, the non-invasive screening methods explored in the present study, which include systemic lupus erythematosus and systemic sclerosis, are of utmost importance.

The present study indicated a strong correlation between the FVC%/DLCO% ratio in pulmonary function tests and mPAP measured by right heart catheterization. The use of FVC%/DLCO% alone for the diagnosis of CTD-PH yielded a high sensitivity (0.794) and specificity (0.789), with an optimal diagnostic threshold of 1.35. The sensitivity for diagnosing PH improved to 0.94 when the aforementioned method was combined with echocardiography; the value obtained was significantly higher than that of echocardiography alone. This combination approach is more favorable for the early detection of CTD-PH. This finding can be explained by the pathological mechanisms of CTD-PH. In pulmonary function tests, FVC primarily reflects the ventilatory function of the patient, while DLCO reflects the diffusion function. Inflammatory stimulation in CTD leads to the excessive proliferation and differentiation of endothelial cells, smooth muscle cells and fibroblasts in pulmonary arteries, resulting in the thickening of the pulmonary arterial intima and decreased peripheral vascular beds, thereby causing a decline in DLCO. By contrast, FVC does not decrease proportionally (9,32,33). Therefore, the FVC%/DLCO% ratio may serve as a diagnostic criterion for CTD-PH. It is recommended that clinicians regularly conduct pulmonary function tests in the routine management of patients with CTD to identify individuals with PH at an early stage and to intervene early, in order to minimize adverse outcomes. The 2022 ESC/ERS guidelines (3) incorporate various indicators, including the 6-min walking distance and plasma BNP, into the risk stratification of PH. However, in the present study, the 6-min walking distance did not exhibit an independent predictive value for PH. It was speculated that this may be due to the potential influence of joint involvement and restricted activity in certain patients with CTD. Plasma BNP was identified as an independent predictive factor for PH; however, no significant correlation was noted between plasma BNP and mPAP (P=0.054). This may be attributed to the relatively small sample size and certain biases in the present study.

Considering that a relatively large proportion of patients with CTD exhibited concomitant ILD with 20.58% (7/34) in the PH group and 10.53% (2/19) in the non-PH group, subgroup analysis was conducted to exclude the influence of decreased DLCO in ILD on the final results. The analysis revealed an optimal correlation between FVC%/DLCO% and mPAP even following the exclusion of patients with ILD.

To the best of our knowledge, the present study is the first to propose the application of the FVC%/DLCO% ratio as a predictive tool for the identification of PH associated with a range of CTDs. However, this was is a single-center study with a relatively small sample size and further clinical validation is required. Further larger multicenter cohort studies are warranted in order to further verify the established threshold of FVC%/DLCO% for the diagnosis of CTD-PH and explore its association with overall disease mortality.

## Figures and Tables

**Table I:** Disease manifestation of CTD-PH.

Classification of CTD-PH	Count, n (%)
Systemic lupus erythematosus	14 (41.2)
Rheumatoid arthritis	6 (17.6)
Mixed connective tissue disease	6 (17.6)
Inflammatory myopathies	3 (8.8)
Systemic sclerosis	3 (8.8)
Sjogren's syndrome	2 (5.9)

CTD, connective tissue disorder-associated pulmonary hypertension.

**Table II:** Comparison of the demographic characteristics and general information of the patients.

Characteristic	PH (n=34)	non-PH (n=19)	P-value
Age (years)	45.85±12.87	41.15±14.78	0.233
Sex (male/female)	2/32	1/18	1.000
BMI (kg/m^2^)	20.72±1.85	20.34±2.5	0.541
Smoking index^a^	26.47±18.55	48.42±33.76	0.537
FVC%/DLCO%	1.54±0.43	1.11±0.33	<0.001
mPAP (mmHg)	38.06±11.25	15.53±3.72	<0.001
Echocardiographic PASP (mmHg)	54.97±18.55	40.26±9.12	<0.001
Plasma BNP (pg/ml)	1,010.72±1,848.83	117.16±138.36	0.008
WBC (x10^9^/l)	5.26±2.26	4.78±2.45	0.505
RDW (%)	28.19±15.85	26.33±12.84	0.643
ESR (mm/h)	32.41±26.87	44.42±26.27	0.122
CRP (mg/l)	15.02±23.15	14.25±18.11	0.902
6MWD (m)	348.71±90.66	431.95±59.26	<0.001

^a^The smoking index is calculated by multiplying the average number of roots per day by the number of years spent smoking. A total of 2 individuals (2/34) in the PH group reported a history of smoking, while 3 individuals (3/19) in the non-PH group reported a history of smoking. BMI, body mass index; FVC, forced vital capacity; DLCO, diffusing capacity of the lungs for carbon monoxide; mPAP, mean pulmonary artery pressure; PASP, pulmonary artery systolic pressure; BNP, brain natriuretic peptide; WBC, white blood cell count; RDW, red cell distribution width; ESR, erythrocyte sedimentation rate; CRP, C-reactive protein; 6MWD, 6-min walk distance.

**Table III:** Multivariate logistic regression analysis related to PH.

Characteristic	OR	95% CI	P-value
FVC%/DLCO%	1.45	1.120-1.890	0.027
Echocardiographic PASP	1.893	1.512-2.482	0.019
Plasma BNP	1.994	1.290-2.399	0.019
6MWD	1.005	0.991-1.019	0.470

PH, pulmonary hypertension; FVC, forced vital capacity; DLCO, diffusing capacity of the lungs for carbon monoxide; PASP, pulmonary artery systolic pressure; BNP, brain natriuretic peptide; 6MWD, 6-min walk distance.

## Data Availability

The datasets used and/or analyzed during the current study are available from the corresponding author on reasonable request.
